# Supplementary material for: The BASP1 transcriptional corepressor modifies chromatin through lipid-dependent and lipid-independent mechanisms
Source: iScience. 2022 Jul 20;25(8):104796. doi: 10.1016/j.isci.2022.104796 (PMC9379585; doi:10.1016/j.isci.2022.104796)
Supplement: Document S1. Figures S1–S3 [file mmc1.pdf]

iScience, Volume *25*

Supplemental information

The BASP1 transcriptional corepressor modifies chromatin through lipid-dependent and lipid-independent mechanisms

Alexander J. Moorhouse, Amy E. Loats, Kathryn F. Medler, and Stefan G.E. Roberts

A

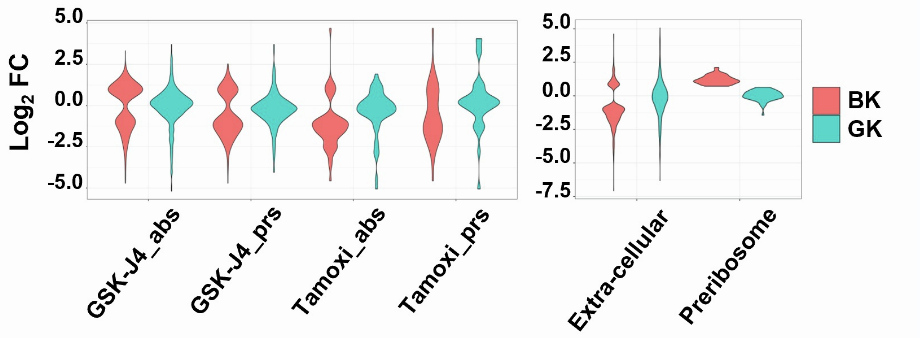

B

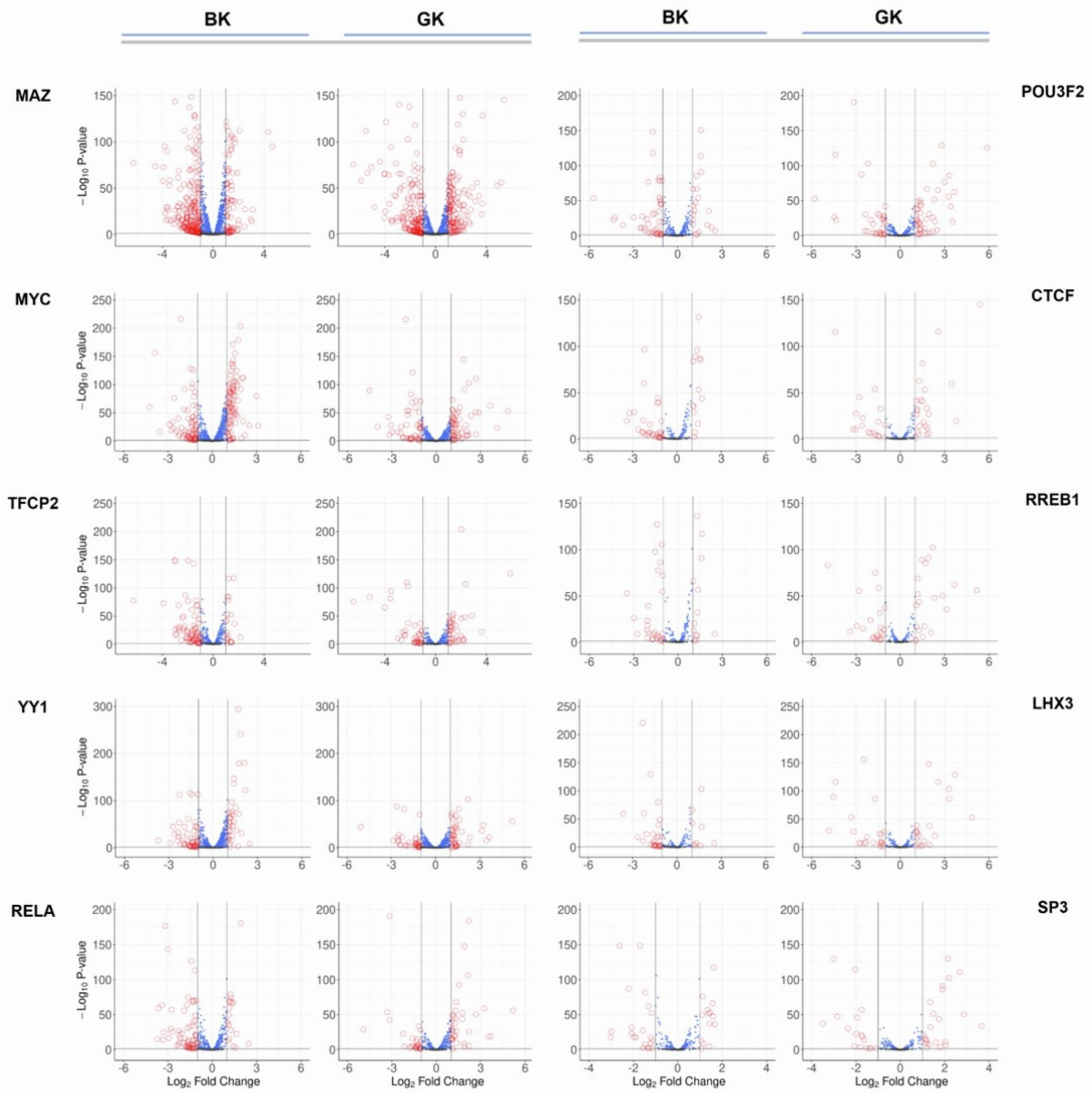

Supplementary Figure 1: Pathways and transcription factors regulated by BASP1. Related to Figure 4. (A) Differential gene expression for genes associated with the absence or deficiency (abs) and presence or abundance (prs) of GSK-J4 and tamoxifen (left panel), and those involved in regulation of the extracellular space and pre-ribosome processes (right panel).
(B) Functional enrichment of transcription factors (TFs) were identified from significantly differentially expressed genes (padj ≤ 0.05, FC ≥ 1, BK vs VK), YY1 and CTCF targets are also included, differential expression of TF target genes is shown in volcano plots for BK and GK.

A

source	term_name	term id	negative log10 of adjusted p value	term size	intersection size	query size	enrichment*
GO:CC	cell periphery	GO:0071944	6	6178	187	400	96.657
GO:CC	membrane	GO:0016020	2	9829	247	400	48.903
GO:BP	developmental process	GO:0032502	3	6678	189	388	45.714
GO:BP	anatomical structure development	GO:0048856	3	6164	178	388	44.394
TF	Factor: OCT-2; motif: ATGMATATGCWAAT	TF:M08836	4	3733	124	422	33.025
TF	Factor: POU2F2; motif: NWTRMATATKCAWN	TF:M04073	3	4201	133	422	30.501
GO:BP	multicellular organism development	GO:0007275	2	5670	162	388	25.847
TF	Factor: HoxA5; motif: NYMATTAN	TF:M10706	3	3573	116	422	22.779
TF	^Factor: Oct-4; motif: ATTGWSWTGCWAAWN	TF:M01124	2	6101	181	450	21.731
GO:BP	regulation of signal transduction	GO:0009966	2	3251	107	388	20.646
GO:CC	cell junction	GO:0030054	4	2107	78	400	20.243
GO:BP	cell differentiation	GO:0030154	2	4392	131	388	17.400
GO:BP	cell surface receptor signaling pathway	GO:0007166	2	3226	104	388	15.580
GO:BP	positive regulation of cell communication	GO:0010647	2	1809	69	388	11.530
GO:BP	^positive regulation of multicellular organismal process	GO:0051240	3	1497	64	415	9.546
GO:BP	plasma membrane bounded cell projection organization	GO:0120036	2	1596	61	388	7.577
GO:BP	neurogenesis	GO:0022008	2	1698	63	388	6.978
GO:BP	biological adhesion	GO:0022610	2	1509	58	388	6.497
GO:CC	cell surface	GO:0009986	2	896	39	400	5.204
GO:BP	regulation of protein kinase activity	GO:0045859	2	809	37	388	3.208
KEGG	Cytokine-cytokine receptor interaction	KEGG:04060	2	293	17	179	2.541
GO:BP	positive regulation of protein serine/threonine kinase activity	GO:0071902	2	317	21	388	1.559

*Enrichment = P x (Ts/Qs). P = negative Log10 adjusted p value. Ts = term size. Qs = query size

B

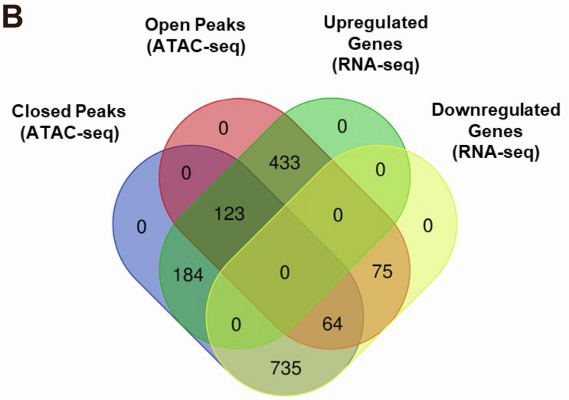

C

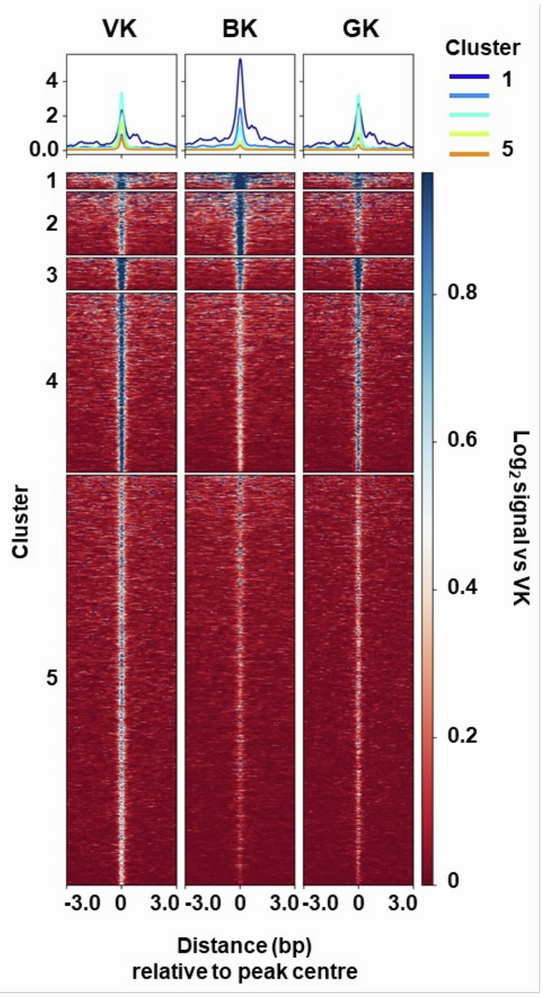

Supplementary Figure 2: Regulation of chromatin accessibility by BASP1 and BASP1 G2A. Related to Figure 5. (A) Table produced from combined g:profiler results for genes (ordered and unordered analysis), and peak regions (unordered analysis only) for down regulated genes with closed chromatin (peaks and genes at padj ≤ 0.05 and FC ≥ 1, BK vs VK) (see Fig 4). Terms shown appeared in ≥ 2 of the three analyses, statistics shown are from unordered peak analysis, except where shown from unordered gene analysis ^. Data are ordered by enrichment score calculated as a function of the negative Log10 adjusted p value and term size to query size ratio. (B) Up and down regulated genes counts at open and closed chromatin regions are shown for differentially expressed peaks and genes at padj ≤ 0.05 and FC ≥ 1 for GK vs VK. (C) Heatmaps showing ATAC signal intensity prepared using deepTools computeMatrix and plotHeatmap with k-means clustering.

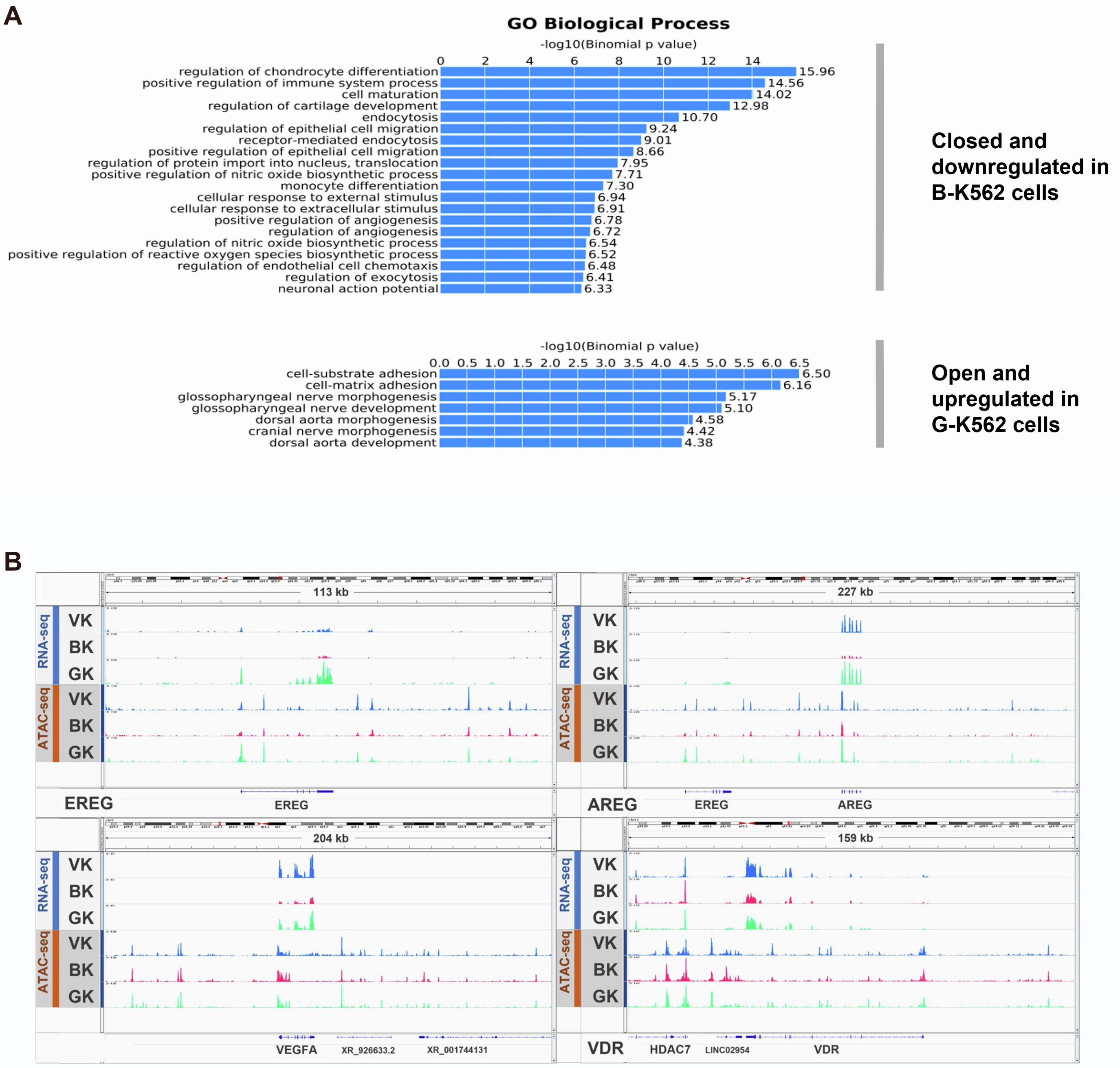

Supplementary Figure 3 : Genes regulated through the modulation of chromatin accessibility by BASP1. Related to Figure 5. (A) Functional enrichment among chromatin regions prepared from corresponding peak sets of venn diagram S2B using the Genomic Regions Enrichment of Annotations Tool (GREAT). (B) Chromatin peaks and gene expression with expanded view compared to Figure 5F are shown for EREG, AREG, VDR and VEGFA.
